# Supplementary material for: The Azotobacter vinelandii AlgU regulon during vegetative growth and encysting conditions: A proteomic approach
Source: PLoS One. 2023 Nov 15;18(11):e0286440. doi: 10.1371/journal.pone.0286440 (PMC10651043; doi:10.1371/journal.pone.0286440)
Supplement: S1 Fig — 126 proteins with altered expression in the absence of the sigma factor AlgU, were analized. Interaction nodes such as those constituted by proteins involved in lipids metabolism (green circle), central metabolism (blue circle), flagella biogenesis and motility (cyan circle), trehalose synthesis (black circle) and enzymes for alginate production (pink circle) are indicated. Disconnected nodes are hided; the network was generated using an interaction score of 0.7. (PDF) [file pone.0286440.s001.pdf]

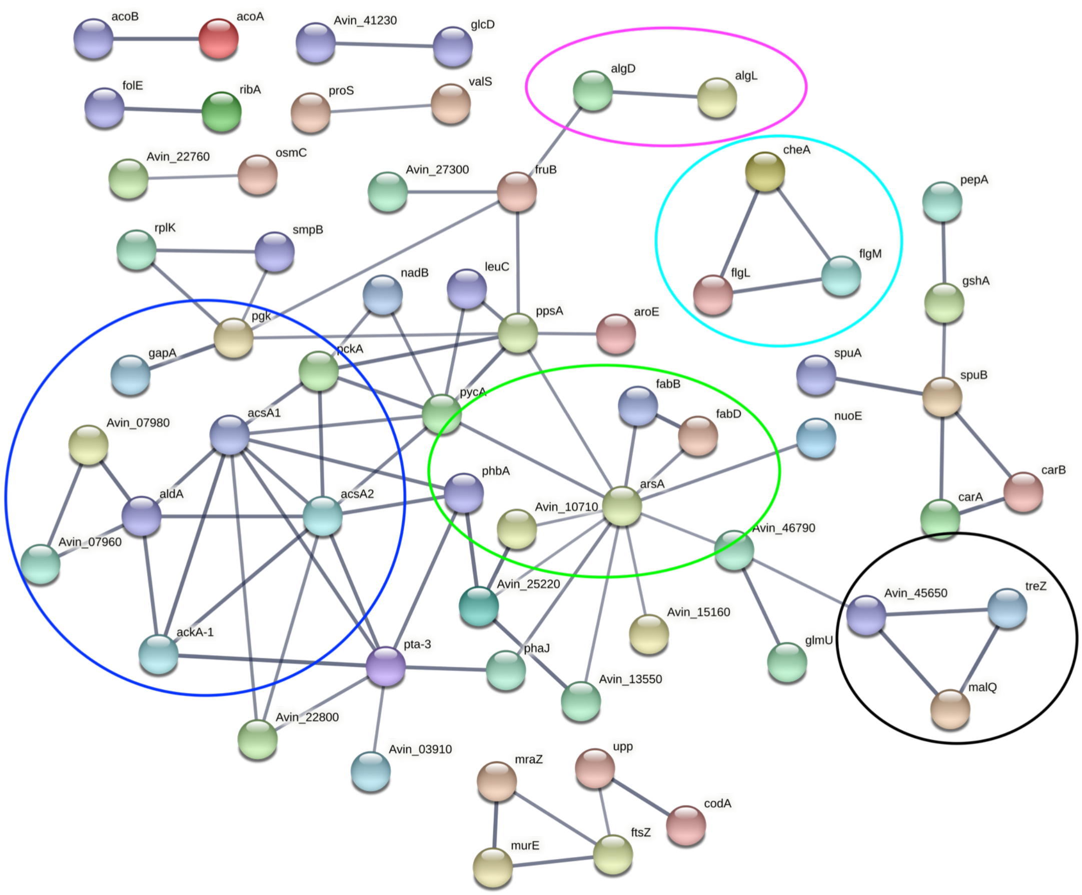

S1 Fig. Visualization of protein-protein interaction network by String of AlgU controled proteins during vegetative conditions. 126 proteins with altered expression during vegetative growing conditions, in the absence of the sigma factor AlgU were analized. Interaction nodes such as those constituted by proteins involved in lipids metabolism (green circle), central metabolism (blue circle), flagella biogenesis and motility (cyan circle), trehalose synthesis (black circle) and enzymes for alginate production (pink circle) are indicated. Disconected nodes are hided; the network was generated using an interaction score of 0.7.
