# Supplementary material for: The Azotobacter vinelandii AlgU regulon during vegetative growth and encysting conditions: A proteomic approach
Source: PLoS One. 2023 Nov 15;18(11):e0286440. doi: 10.1371/journal.pone.0286440 (PMC10651043; doi:10.1371/journal.pone.0286440)
Supplement: S2 Fig — 305 down-represented proteins in the absence of the sigma factor AlgU, during encysting conditions were analized. Interaction nodes such as those constituted by proteins involved in ribosome assembly (red circle), nitrogen fixation (blue circle), amino acid metabolism (cyan circle), respiration (black circle), central metabolism (green circle) and enzymes for alginate (pink circle) or trehalose (yellow circle) production are indicated. Disconnected nodes are hided; the network was generated using an interaction score of 0.7. (PDF) [file pone.0286440.s002.pdf]

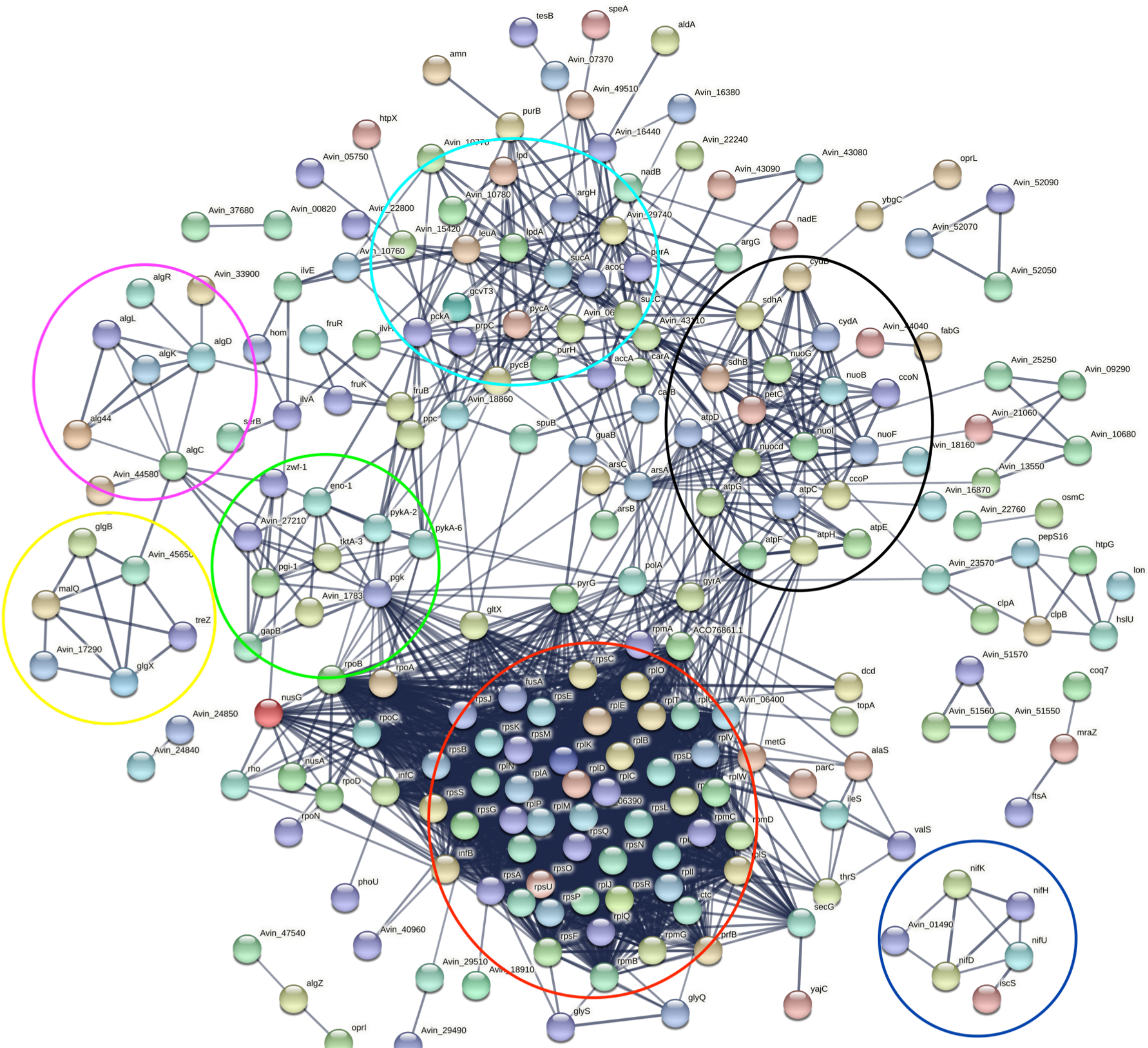

S2 Fig. Visualization of protein-protein interaction network generated by String of proteins positively controled by AlgU during encystment. 305 down-represented proteins in the absence of the sigma factor AlgU, during encysting conditions were analyzed. Interaction nodes such as those constituted by proteins involved in ribosome assembly (red circle), nitrogen fixation (blue circle), amino acid metabolism (cyan circle), respiration (black circle), central metabolism (green circle) and enzymes for alginate (pink circle) or trehalose (yellow circle) production are indicated. Disconected nodes are hided; the network was generated using an interaction score of 0.7.
