# Supplementary material for: The Azotobacter vinelandii AlgU regulon during vegetative growth and encysting conditions: A proteomic approach
Source: PLoS One. 2023 Nov 15;18(11):e0286440. doi: 10.1371/journal.pone.0286440 (PMC10651043; doi:10.1371/journal.pone.0286440)
Supplement: S3 Fig — 184 up-regulated proteins in the absence of the sigma factor AlgU, during encysting conditions were analyzed. Interaction nodes such as those constituted by proteins involved in amino acids (green circle), aminoacyl-tRNA (cyan circle), or fatty acid (black circle) biosynthesis are indicated. Disconnected nodes are hided; the network was generated using an interaction score of 0.7. (PDF) [file pone.0286440.s003.pdf]

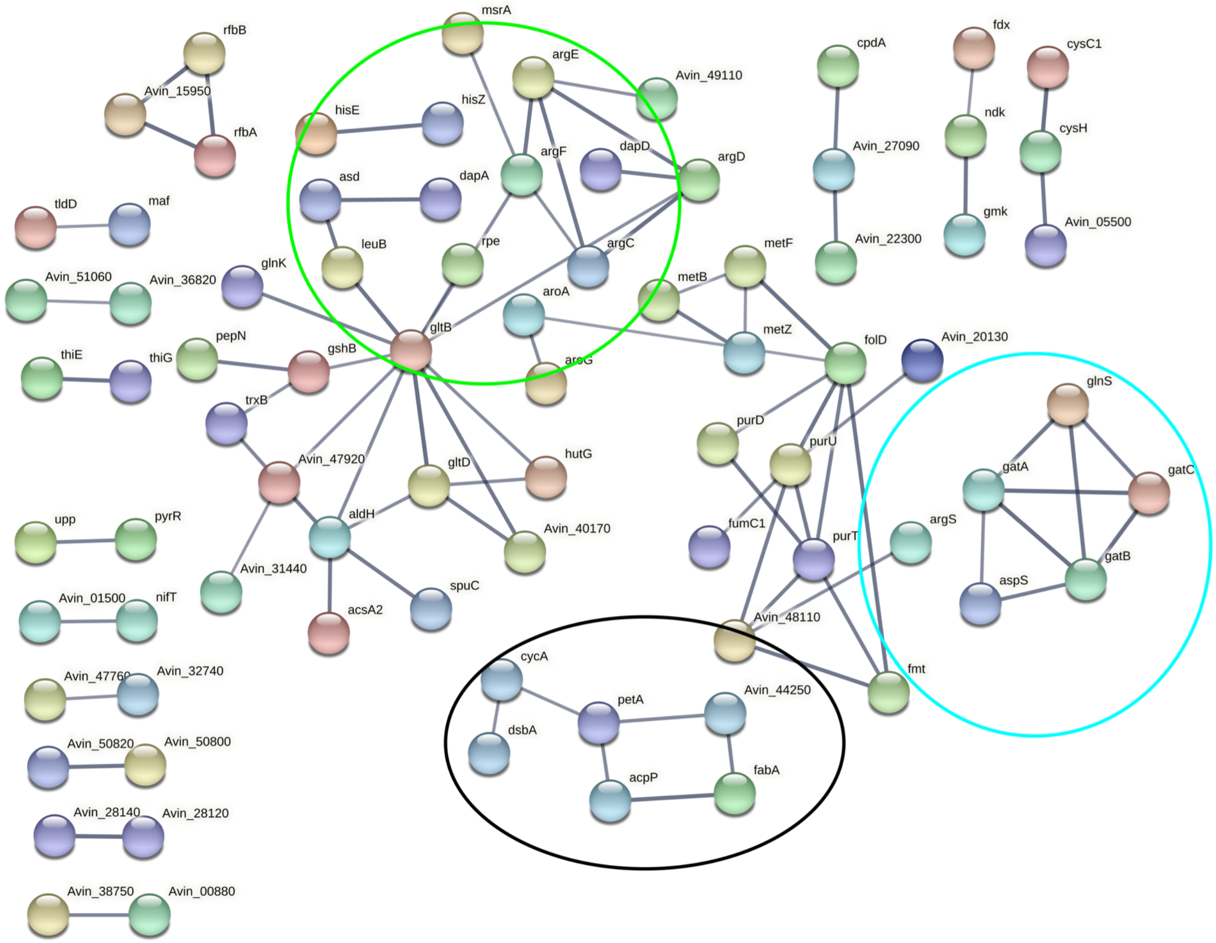

S3 Fig. Visualization of protein-protein interaction network by String of proteins negatively controled by AlgU. The 184 up-regulated proteins in the absence of the sigma factor AlgU, during encysting conditions were analyzed. Interaction nodes such as those constituted by proteins involved in amino acids (green circle), aminoacyl-tRNA (cyan circle), or fatty acid (black circle) biosynthesis are indicated. Disconected nodes are hided; the network was generated using an interaction score of 0.7.
