# Supplementary material for: The Azotobacter vinelandii AlgU regulon during vegetative growth and encysting conditions: A proteomic approach
Source: PLoS One. 2023 Nov 15;18(11):e0286440. doi: 10.1371/journal.pone.0286440 (PMC10651043; doi:10.1371/journal.pone.0286440)
Supplement: S4 Fig — A Position-Specific Scoring Matrix was used to identify AlgU binding motifs in the genome of P. fluorescens (A) or A. chroococcum (B) (see Materials and Methods section for details). The predicted AlgU binding motifs with are shown with 16 (upper panels) or 17 (lower panels) bp spacers between the -10 and -35 boxes. (PDF) [file pone.0286440.s004.pdf]

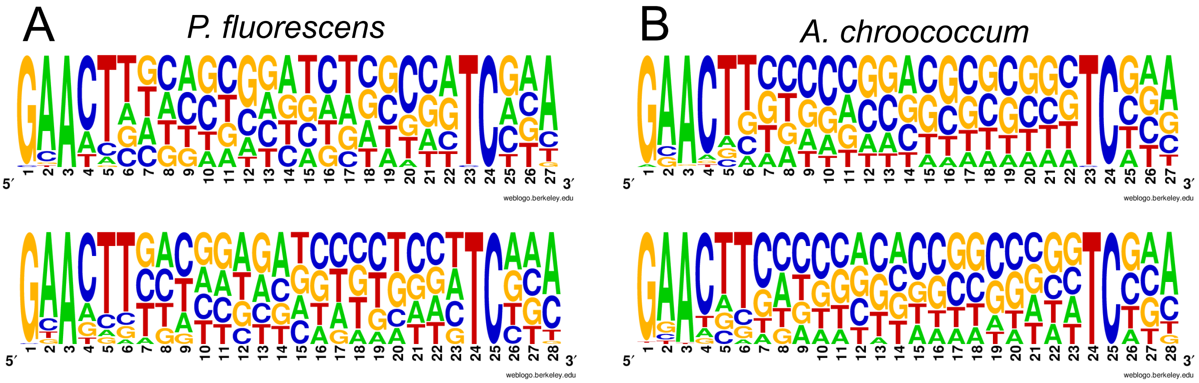

S4 Fig. AlgU sigma factor binding motifs. A Position-Specific Scoring Matrix was used to identify AlgU binding motifs in the genome of *P. fluorescens* (A) or *A. chroococcum* (B) (see Materials and Methods section for details). The predicted AlgU binding motifs with spacers of 16 (upper panels) or 17 (lower panels) bp between the -10 and -35 boxes are shown.
